# Altered cytokine and chemokine profile linked to autoantibody and pathogen reactivity in mothers of autistic children

**DOI:** 10.3389/fpsyt.2024.1348092

**Published:** 2024-05-22

**Authors:** Janna McLellan, Lisa Croen, Ana-Maria Iosif, Cathleen Yoshida, Paul Ashwood, Robert H. Yolken, Judy Van de Water

**Affiliations:** ^1^ Department of Internal Medicine, Division of Rheumatology, Allergy, and Clinical Immunology, University of California, Davis, CA, United States; ^2^ Kaiser Permanente Research Division, Oakland, CA, United States; ^3^ Department of Public Health Sciences, University of California, Davis, CA, United States; ^4^ MIND Institute, University of California, Davis, CA, United States; ^5^ Department of Medical Microbiology and Immunology, University of California, Davis, CA, United States; ^6^ Johns Hopkins School of Medicine, Johns Hopkins University, Baltimore, MS, United States

**Keywords:** cytokine, chemokine, autoantibody, autism, autoimmunity

## Abstract

Maternal autoimmunity, and more specifically, the production of specific maternal autoantibodies, has been associated with altered offspring neurodevelopment. Maternal autoantibody-related (MAR) autism is a subtype of autism that is linked to gestational exposure to certain combinations of autoantibodies to proteins known to be important for fetal neurodevelopment. We wanted to address whether mothers with autism-specific patterns of autoantibodies have a skewed cytokine and chemokine profile during an immune response to infection. To do so, we examined a subset of mothers from the Early Markers for Autism (EMA) study who either produced known patterns of MAR autoantibodies (MAR+) or did not (MAR-). We compared the cytokine/chemokine profiles of MAR+ and MAR- mothers in the context of positive immunoglobulin G (IgG) reactivity to several viral and parasitic agents. We observed that MAR+ mothers have a higher level of proinflammatory cytokine interferon-gamma regardless of IgG status. Additionally, when comparing MAR+ and MAR- mothers in the context of the different pathogens, MAR+ mothers consistently had increases in multiple proinflammatory cytokines and chemokines.

## Introduction

Several identified maternal factors increase offspring’s risk of neurodevelopmental disorders, including autism. Among these include maternal obesity (1), maternal immune activation (MIA) (2, 3), infection during pregnancy, and maternal autoimmunity and immune dysregulation (4, 5). In particular, the production of specific maternal autoantibodies (ABs) is associated with a subtype of autism known as maternal autoantibody-related (MAR) autism. These ABs target proteins in the brain and the periphery that are known to be important for proper neurodevelopment (6, 7). The eight proteins are collapsin response mediator proteins 1 and 2 (CRMP1/2), neuron-specific enolase (NSE), lactate dehydrogenase A and B (LDHA/B), stress-induced phosphoprotein 1 (STIP1), guanine deaminase (GDA), and Y-box binding protein 1 (YBOX-1) (6–8). The passage of maternal IgG to the fetus is an essential protective mechanism during pregnancy (9, 10). Thus, it is believed that the MAR ABs are also able to cross the placenta, where they can interact with their targets in the fetal compartment, leading to the observed autism pathology (11, 12).

Clinical studies have shown that only specific patterns of MAR ABs are highly associated with an autism outcome; these AB patterns include CRMP1+GDA, CRMP1+CRMP2, CRMP2+STIP1, CRMP1+STIP1, GDA+YBOX1, STIP1+NSE, and LDHA/B+YBOX1 (13, 14). Moreover, these investigations have revealed that the offspring of mothers with MAR have higher ADOS scores, indicating more severe symptoms. Additionally, specific MAR patterns are associated with more severe clinical outcomes, with maternal ABs to CRMP1+CRMP2 being linked to more severe developmental delay (13, 15). Preclinical studies using rodent models that replicate the clinical MAR AB exposure have identified several behavioral and neurological implications of AB exposure. More specifically, rodent offspring gestationally exposed to a combination of LDHA/B+CRMP1+STIP1, the first clinically observed MAR pattern (7), displayed decreases in social behavior and increases in species-specific anxiety behaviors (11, 16). In addition, we observed AB deposition in the early postnatal brains of these offspring and volumetric changes in several brain regions implicated in ASD pathology (11).

While it remains unclear what triggers some women to produce ABs to these proteins, skewing of the maternal cytokines and chemokines in mothers who produce MAR ABs has been documented (McLellan et al., Manuscript Accepted). Thus, these mothers could have an underlying immune dysregulation, increasing their susceptibility to loss of self-tolerance. Here, we sought to determine if mothers with circulating MAR-specific patterns of ABs during pregnancy also have skewed cytokine and chemokine profiles when mounting an immune response to viral and parasitic agents. To do so, we used participants from the Early Markers for Autism (EMA) study who were exposed to and had detectable immunoglobulin G (IgG) levels to either cytomegalovirus (CMV), Epstein-Barr virus (EBV), influenza type A (Flu-A), varicella-zoster virus (VZV), herpes-simplex I virus (HSV), toxoplasma gondii (TOXO), or who tested positive for c-reactive protein (CRP) between 15 and 19 weeks gestation. We then examined the differential immune response to pathogen exposure between mothers with MAR-specific ABs and those without by assessing their mid-gestational cytokines and chemokines levels. Understanding the level of the cytokine/chemokine response to pathogens in mothers who are positive for MAR ABs will provide additional information regarding the potential mechanistic foundation underlying the autoimmunity noted in these mothers, which can increase the risk of autism in their children.

## Methods

### Participants

All participants were enrolled in the EMA study (17). Expanded details on the study population have been previously published (18, 19). Mothers were eligible for inclusion in EMA if they participated in the prenatal expanded alpha-fetoprotein screening program (XAFP) and delivered a live-born infant between July 2000 and September 2003. The original study was comprised of three groups: children with autism (n=486), children with developmental delay (DD) but not autism (n=174), and general population controls (n=397) and their respective mothers. The California Department of Developmental Services assessed children with autism and DD, and their diagnostic status was validated by a blinded clinician using the Diagnostic and Statistical Manual of Mental Disorders, Fourth Edition (DSM-IV) criteria. From the original EMA participants, we first identified all MAR+ mothers, being those with at least one of the MAR autism-specific patterns of ABs, including CRMP1+GDA, CRMP1+CRMP2, CRMP2+STIP1, CRMP1+STIP1, GDA+YBOX1, STIP1+NSE, and LDHA/B+YBOX1, who had a child with autism (n=37). We then selected a subset for MAR- mothers, those that did not have a MAR autism-specific AB pattern, who had a child with autism (n=37) matching them on maternal age, race/ethnicity, and birthplace to MAR+ mothers. MAR+ and MAR mothers were then tested for reactivity to CMV, EBV, Flu-A, VZV, HSV, TOXO, or CRP using the same mid-gestational blood sample. MAR+ mothers of children who were initially diagnosed with DD and were then reevaluated and determined to have autism were not included (n=6). The institutional review boards of the California Health and Human Services Agency and Kaiser Permanente Northern California approved all study procedures.

### Sample collection

Maternal samples were collected between 15 and 19 weeks of gestation as part of routine prenatal XFAP screening. Samples were collected in serum separator tubes by obstetrical care providers and underwent XFAP testing. Leftover specimens were shipped on dry ice to our laboratory and stored at -80°C before use in maternal autoantibody, IgG against pathogenic antigens, and cytokine/chemokine measurement assays. Expanded details have been previously described (19).

### Measurement of maternal autoantibodies

The presence of maternal ABs to our proteins of interest, CRMP1/2, STIP1, LDHA/B, NSE, YBOX1 and GDA, in this study population, was detected by enzyme-linked immunosorbent assay (ELISA) as previously published (13). Briefly, microtiter plates were coated with antigen (2-3μg/μl as optimized for each protein) diluted in carbonate coating buffer (pH 9.6) and incubated overnight at 4°C. The following day, diluted plasma samples were tested in duplicate. For colorimetric detection, a secondary antibody (goat anti-human IgG-HRP (Kirkegaard & Perry Laboratories, Inc., Gaithersburg, MA)) was used, followed by the addition of BD optEIA liquid substrate for ELISA (BD Biosciences, San Jose, CA). Following incubation, the reaction was stopped with 2N HCl. Absorbance was measured at 450-595 nm using an iMark Microplate Absorbance Reader (Biorad, Hercules, CA, USA). Expanded assay details have been previously described (13).

### Detection of IgG against pathogenic antigens and auto-antigens

Maternal IgG to each agent was measured using solid-phase immunoassay (ELISA) as previously described (20). IgG levels were expressed as μg/ml, and results were not corrected for total protein. CRP levels were measured using the High Sensitivity Human C-Reactive protein kit (IBL America, Minneapolis, Minnesota). Further details have been previously described (21–23).

### Measurement of maternal cytokines and chemokines

The cytokine and chemokine measurements were performed as originally published (19). Mid-gestational serum concentrations of 22 cytokines and chemokines were measured using a Millipore Multiples bead-based kit (Milliplex MAP Human Cytokine/Chemokine Kit; Millipore, Billerica, MA, USA). Further details on assay methods are in the original study publication (19).

### Statistical analysis

Before analysis, all cytokine/chemokine data were natural log-transformed. Descriptive statistics were used to summarize the clinical and socio-demographic variables and the cytokine/chemokine concentrations. Differences between MAR groups were assessed via Chi-square tests for categorical socio-demographic variables and exact Wilcoxon rank-sum tests for continuous socio-demographic variables and IgG values. Multiple linear regression models were used to evaluate the effect of MAR status on cytokine and chemokine levels in mothers with IgG reactivity to at least one of the agents tested. Separate models were run for each cytokine/chemokine with the MAR status (MAR+ or MAR-) as the primary predictor and including maternal race and ethnicity as covariates. In secondary analyses, we restricted the samples to the MAR+ and MAR- mothers who tested positive for that specific infectious agent. We examined each infectious agent separately and evaluated unadjusted MAR+ vs. MAR- differences in each cytokine and chemokine levels using exact Wilcoxon rank-sum tests.

For each analysis, we used false discovery rate (FDR) correction for multiple comparisons to help mitigate the risk of Type I error. We also present the raw *p*-values to provide initial insights into potential associations or differences without adjustments.

Summaries of maternal cytokines, including the mean values and upper and lower quartiles for MAR+ and MAR- mothers and for MAR+ and MAR- mothers in each IgG reactivity group, are shown in **Supplementary Tables 1**-**8**.

## Results

### Differences in IgG reactivity based on MAR AB status

Upon initial assessment of IgG reactivity to infectious agents based on MAR AB status, we identified a higher percentage of MAR+ mothers that had reactivity to CRP, EBV, HSV, and Influenza-A than MAR- mothers. Additionally, MAR+ mothers had significantly higher mean toxoplasmosis IgG levels than MAR- mothers (**Table 1**).

**Table 1:** Demographic information on study participants who were a subset of mothers from the original Early Markers for Autism (EMA) study.

Characteristic	MAR+ (n=37)	MAR- (n=37)	*p-*value^a^
Maternal Birth Country, n (%)			1.00
**US**	6 (16%)	6 (16%)	
**Mexico**	13 (35%)	13 (35%)	
**Other**	18 (49%)	18 (49%)	
Maternal Race, n (%)			0.93
**Caucasian**	22 (60%)	22 (60%)	
**Asian**	10 (27%)	9 (24%)	
**Other**	5 (13%)	6 (16)	
Maternal Ethnicity, n (%)			1.00
**Hispanic**	11 (30%)	11 (30%)	
**Non-Hispanic**	26 (70%)	26 (70%)	
Maternal Age (years), mean (SD)	29.2 (5.3)	29.7 (6.3)	0.95
Maternal Weight at Blood Draw (lbs), mean (SD)	156.8 (45.9)	149.4 (39.6)	0.62
Gestational Age at Blood Draw (days), mean (SD)	117.3 (8.0)	119.3 (8.8)	0.35
IgG Value, mean (SD)			
**Cytomegalovirus** ^b^	0.81 (0.49)	0.65 (0.55)	0.15
**Epstein-Barr Virus** ^c^	0.41 (0.25)	0.37 (0.28)	0.18
**Herpes Simplex Virus**	2.11 (1.23)	1.75 (1.33)	0.17
**Influenza A** ^c^	1.04 (0.33)	0.88 (0.32)	0.054
**Varicella-Zoster Virus**	1.94 (0.49)	1.87 (0.63)	0.80
**Toxoplasmosis** ^d^	0.29 (0.49)	0.22 (0.35)	**0.047**
**C-Reactive Protein** ^c^	1.30 (0.89)	1.43 (0.82)	0.31

MAR+, mothers with MAR-autism specific patterns of autoantibodies; MAR-, mothers without autoantibodies to any of our tested antigens; SD, standard deviation.

^a^Group differences were assessed using chi-square tests for categorical variables and exact Wilcoxon rank-sum test for continuous variables.

Data missing for: -; ^b^2 mothers in the MAR- group; ^c^1 mother in the MAR- group; ^d^1 mother in the MAR+ group and 2 in MAR-.

Bolding indicates IgG values for which the group differences significantly differed from 0 (p<0.05 without correcting for multiple comparisons).

### Cytokine and chemokine profiles in MAR+ and MAR- mothers

We first wanted to determine if, in this sample population, there were differences between the MAR+ and MAR- mothers irrespective of IgG reactivity to any of the infectious agents. When comparing these two groups, we found that MAR+ mothers had higher pro-inflammatory cytokine interferon-gamma levels (IFNγ) levels (**Table 2**). However, this result did not survive multiple comparison adjustments.

**Table 2:** Adjusted cytokine and chemokine differences between MAR+ and MAR- mothers.

Cytokine/Chemokine	MAR+ versus MAR-		
	Estimate	SE	*p-*value
**Eotaxin**	0.35	0.39	0.38
**GM-CSF**	0.34	0.62	0.59
**IFNγ**	0.79	0.35	**0.03**
**IL-2**	0.56	0.40	0.16
**IL-4**	-0.12	0.19	0.56
**IL-6**	0.62	0.54	0.25
**IL-7**	-0.05	0.30	0.87
**IL-8**	0.23	0.40	0.56
**IL-10**	0.43	0.47	0.36
**IL-13**	0.20	0.54	0.71
**IL-17**	0.30	0.57	0.60
**IL-12p40**	0.56	0.50	0.27
**IL-12p70**	0.36	0.38	0.35
**IL-1α**	0.49	0.48	0.32
**IL-1β**	0.63	0.58	0.28
**IL-1Ra**	0.59	0.32	0.07
**IP-10**	0.02	0.19	0.93
**MCP-1**	0.05	0.20	0.82
**MIP-1α**	0.14	0.60	0.81
**MIP-1β**	0.43	0.36	0.24
**TNFα**	0.27	0.27	0.33
**sIL-2Ra**	0.12	0.33	0.73

MAR+, mothers with MAR-autism specific patterns of autoantibodies; MAR-, mothers without autoantibodies to any of our tested antigens; SE, standard error.

^a,b^Estimates represent adjusted differences between cytokines/chemokines concentrations between MAR+ mothers (n=37) and MAR-mothers (n=37) from multiple linear regression models fitted to natural log-transformed neonatal cytokine/chemokine concentrations. The models included a term for maternal MAR status and were adjusted for maternal race and ethnicity.

Bolding indicates cytokines/chemokines for which the adjusted group differences significantly differed from 0 (p<0.05 without correcting for multiple comparisons).

### Cytokine and chemokine profiles in MAR+ and MAR- mothers with IgG reactivity to infectious agents

We next wanted to determine if, in the mothers who had IgG antibodies to either CMV, EBV, HSV, Flu-A, TOXO, VZV, or had positive CRP reactivity, there was a difference in the cytokines/chemokines levels in those that were MAR+ compared to those who were MAR-. To do so, we compared only the MAR+ and MAR- mothers who had positive reactivity to each agent (**Table 3**).

**Table 3:** Cytokine and chemokine differences between MAR+ versus MAR- mothers who had positive reactivity to CMV, EBV, Flu-A, VZV, HSV, or a positive CRP test.

Cytokine/ Chemokine	CMV Positive MAR+ n=12 MAR- n=10			EBV Positive MAR+ n=9 MAR- n=7			Flu-A Positive MAR+ n=14 MAR- n=7			VZV Positive MAR+ n=18 MAR- n=18			HSV Positive MAR+ n=26 MAR- n=21			CRP Positive MAR+ n=10 MAR- n=12		
	Est.	SE	*p*	Est.	SE	*p*	Est	SE	*p*	Est.	SE	*p*	Est.	SE	*p*	Est.	SE	*p*
**Eotaxin**	1.50	0.57	**0.02**	0.31	0.65	0.64	2.13	0.67	**0.005**	0.22	0.51	0.66	0.03	0.52	0.96	1.69	0.74	**0.03**
**GM-CSF**	0.83	1.45	0.57	0.52	1.49	0.73	0.99	1.21	0.43	0.55	0.91	0.55	0.17	0.80	0.84	0.65	1.34	0.63
**IFNγ**	0.35	0.43	0.42	1.16	0.30	0.002^*^	1.54	0.64	**0.03**	0.17	0.50	0.74	1.08	0.42	**0.01**	0.40	0.72	0.58
**IL-2**	0.85	0.67	0.22	-0.13	0.63	0.84	1.84	0.88	**0.05**	0.52	0.65	0.42	0.55	0.52	0.30	1.88	0.74	**0.02**
**IL-4**	-0.02	0.29	0.94	-0.11	0.43	0.80	0.35	0.33	0.30	-0.09	0.24	0.73	-0.20	0.23	0.37	0.04	0.33	0.90
**IL-6**	-0.30	1.02	0.77	1.40	1.25	0.28	0.24	1.07	0.82	0.15	0.73	0.84	0.67	0.77	0.39	0.05	0.96	0.96
**IL-7**	0.28	0.42	0.51	0.18	0.61	0.77	0.15	0.48	0.75	-0.22	0.50	0.66	-0.03	0.43	0.95	0.35	0.56	0.54
**IL-8**	-0.07	0.52	0.89	0.64	0.97	0.52	-0.09	0.84	0.91	-0.63	0.54	0.25	0.48	0.53	0.36	0.46	0.58	0.44
**IL-10**	0.66	1.21	0.59	0.75	1.36	0.59	0.40	0.95	0.68	0.29	0.63	0.65	0.34	0.64	0.60	0.01	0.76	0.99
**IL-13**	0.45	1.00	0.66	2.49	1.03	**0.03**	0.20	0.98	0.84	-0.10	0.74	0.90	-0.19	0.66	0.77	1.10	1.02	0.29
**IL-17**	0.54	0.95	0.58	0.78	1.17	0.51	2.22	1.08	0.055	-0.06	0.80	0.94	0.31	0.69	0.65	0.32	1.06	0.77
**IL-12p40**	0.77	1.01	0.46	0.90	0.79	0.28	0.52	1.07	0.63	0.15	0.76	0.85	0.43	0.67	0.53	2.08	1.03	0.06
**IL-12p70**	-0.09	0.73	0.91	0.06	0.68	0.94	0.51	0.91	0.58	0.39	0.62	0.54	0.06	0.50	0.91	0.76	0.85	0.38
**IL-1α**	0.43	0.84	0.62	0.86	0.99	0.40	0.31	0.79	0.70	-0.13	0.63	0.84	0.08	0.58	0.89	0.66	0.88	0.47
**IL-1β**	0.20	1.23	0.88	0.43	1.42	0.77	0.35	1.22	0.77	0.19	0.76	0.80	0.95	0.86	0.28	-0.06	0.91	0.95
**IL-1Ra**	0.74	0.62	0.25	0.81	0.76	0.30	0.32	0.55	0.57	0.87	0.44	0.06	0.55	0.43	0.20	0.74	0.54	0.18
**IP-10**	0.12	0.32	0.72	-0.05	0.23	0.83	-0.19	0.47	0.69	0.22	0.32	0.50	0.12	0.25	0.63	0.35	0.41	0.39
**MCP-1**	0.10	0.42	0.82	0.19	0.48	0.69	0.09	0.41	0.83	-0.01	0.29	0.97	0.03	0.30	0.93	-0.46	0.32	0.17
**MIP-1α**	-0.11	1.13	0.92	0.50	1.62	0.76	0.86	1.11	0.45	0.15	0.77	0.85	0.40	0.86	0.64	0.27	0.95	0.78
**MIP-1β**	0.05	0.75	0.95	0.31	0.92	0.74	1.16	0.67	0.10	0.59	0.42	0.16	0.57	0.53	0.29	0.79	0.45	0.09
**TNFα**	0.09	0.56	0.87	0.06	0.72	0.93	0.46	0.45	0.32	0.15	0.34	0.66	0.16	0.41	0.70	-0.13	0.41	0.75
**sIL-2RA**	-0.03	0.91	0.97	0.31	1.01	0.76	0.12	0.79	0.88	-0.19	0.57	0.74	0.32	0.41	0.44	0.37	0.45	0.42

Bolding indicates significant cytokines/chemokines for which the group differences significantly differed from 0 (p<0.05 without correcting for multiple comparisons).

MAR+, mothers with MAR-autism specific patterns of autoantibodies; MAR-, mothers without autoantibodies to any of our tested antigens; CMV, Cytomegalovirus; EBV, Epstein-Barr virus; Flu-A, Influenza A virus; VZV, Varicella-Zoster virus; HSV, Herpes Simplex Virus; CRP, C-reactive protein; SE, standard error.

^*^Group differences remained significant after correcting for multiple comparisons using false discovery rate.

Overall, MAR+ mothers had higher levels of cytokines and chemokines in the presence of reactivity to pathogenic agents. Eotaxin (CCL11) was significantly higher in MAR+ mothers compared to MAR- mothers in the presence of reactivity to CMV, Flu-A, and CRP. IFNγ was significantly higher in MAR+ mothers versus MAR- mothers in the presence of reactivity to EBV, Flu-A, and HSV. MAR+ mothers who were reactive to Flu-A and CRP also had significantly higher levels of the T-cell-stimulating cytokine IL-2 than MAR- mothers. In addition, MAR+ mothers with reactivity to EBV had significantly higher levels of the T-helper type II (Th2) cytokine IL-13 compared to MAR- mothers with EBV reactivity. There were no statistically significant differences between MAR+ and MAR- mothers with reactivity to VZV. Due to the small sample size (n=3 per group), differences between MAR+ and MAR- mothers with reactivity to toxoplasmosis were not assessed. When we adjusted for maternal race and ethnicity, MAR+ mothers with reactivity to EBV showed significantly higher levels of eotaxin (*p*=0.007), MAR+ mothers with reactivity to VZV had higher IL-1Ra (*p*=0.03), and MAR+ mothers with reactivity to CRP had higher IL-12p40 (*p*=0.04) compared to MAR- mothers. After controlling for multiple comparisons, only IFNγ remained significantly different (FDR adjusted *p*=0.04) between MAR+ and MAR- who also had reactivity to EBV.

## Discussion

Maternal ABs to CRMP1/2, STIP1, LDHA/B, Y-BOX1, GDA, and NSE have been linked to a subtype of autism known as MAR autism (6–8). More specifically, certain MAR-AB patterns have been shown to be highly associated with an autism outcome (13, 14). It is unclear whether some women are susceptible to the production of these ABs due to underlying immune dysregulation or if their autoimmune status is inherently associated with changes in other arms of the immune system, such as cytokine and chemokine production. In addition to autoantibodies, excessive activation of the maternal immune system during pregnancy has frequently been associated with an increased risk of offspring development of autism (2, 3). Thus, understanding the immune status in the context of infection in mothers with MAR+ ABs provides valuable insight into the immune dysregulation that could underlie the loss of self-tolerance, resulting in the production of MAR-ABs.

Understanding the maternal gestational immune status in the context of infection for mothers with MAR+ ABs provides valuable insight into the immune dysregulation that could underlie the loss of self-tolerance. Herein, we used this strategy to examine a sub-population of participants enrolled in the EMA study to determine if MAR+ mothers had changes in their cytokine/chemokine profile when they also had IgG reactivity to infectious agents or tested positive for C-reactive protein when compared to those mothers who had the same infection status but were MAR-.

Regardless of IgG reactivity, MAR+ mothers had higher levels of the proinflammatory cytokine IFNγ, suggesting an underlying inflammatory state in these mothers. Co-stimulatory activity, which the presence of inflammatory cytokines can induce, is necessary for the adaptive immune system to respond to antigens and, therefore, generate antibodies. In the absence of co-stimulation, immune tolerance is induced (24). Thus, an underlying inflammatory state could predispose MAR+ mothers to their autoimmune state.

When we considered MAR+ mothers with IgG reactivity to different infectious agents, including EBV, HSV, and Flu-A, we noted an increase in serum levels of several proinflammatory cytokines, including IFNγ. For example, MAR+ mothers with IgG reactivity to CMV and Flu-A also had increased levels of the proinflammatory chemokine eotaxin. While important for recruiting eosinophils and T cells to sites of infection, excess eotaxin has been associated with allergic and autoimmune diseases, suggesting negative implications, such as increased risk for autoimmune-related health consequences, for MAR+ mothers (25, 26). Further, higher levels of neonatal eotaxin in newborn bloodspots have been previously associated with an increased risk of autism (18). Interestingly, IL-13 was higher in MAR+ mothers with IgG reactivity for EBV. IL-13 functions to promote proliferation of B cells and regulate eosinophilic inflammation. However, previous studies have shown that IL-13 production is induced in B cells early during EBV infection via the EBV protein Zta, contributing to the proliferation of EBV-infected B cells (27). This could suggest that MAR+ mothers may have more dramatic effects of EBV-associated immune dysregulation. In addition to their AB status, higher levels of inflammation in mothers during gestation can also have lasting impacts on their offspring. Therefore, we also assessed differences in CRP reactivity between MAR+ and MAR- mothers. MAR+ mothers, who had elevated CRP levels, which is used as a marker of inflammation, had significantly higher levels of the proinflammatory Th-1 cytokine IL-2 and eotaxin than CRP+ MAR- mothers.

Taken together, these data indicate that MAR+ mothers are susceptible to higher levels of inflammation during the response to infectious agents and in the absence of infection. Additionally, dysregulation in key cytokines/chemokines in the context of infection suggests a suboptimal immune response, which may predispose MAR+ mothers to a loss of self-tolerance and reactivity to MAR-ABs.

Our analyses should be considered in light of several study limitations. First, as we only measured the mothers’ maternal cytokines/chemokines and IgG status at one mid-gestational timepoint, we do not have insight into how the cytokines/chemokines may have changed throughout pregnancy. Second, the infectious agents against which we assessed reactivity can remain latent in the body. Therefore, IgG reactivity for these agents can only indicate infection at some time but will not indicate if there is an active infection during pregnancy. In addition, as pregnancy is a unique immune state in which particular responses may be dampened, IgG levels during pregnancy could differ from pre- to post-pregnancy. However, this is less likely given that the immune response during pregnancy skews toward antibody production and away from a cellular response (28). Third, as this analysis only included 37 MAR+ and 37 MAR- mothers and only some had IgG reactivity to the different infectious agents, our sample size in these comparisons was very small. Due to the modest sample size in our study, the statistical power was limited, and we elected to present both raw *p*-values and the results after corrections for multiple comparisons, offering a comprehensive view of the statistical analysis. As a result, it is important to exercise caution when interpreting *p*-values that are not adjusted for multiple comparisons, as they may lead to an increased risk of Type I errors. Thus, our findings in this preliminary study should be regarded as tentative. However, given the potential for broader clinical implications, we hope they suggest possible directions for future targeted research studies. A larger study would likely yield more robust results and be adequately powered to examine associations in the presence of multiple confounders and covariates. Future studies to address how MAR AB production is related to the response to active infection in a larger sample cohort will help to address the overall immune state of mothers possessing these autism-specific ABs. Finally, as we only considered mothers of children who were later diagnosed with autism, we cannot assess how the maternal levels of cytokines/chemokines in the context of IgG reactivity to infectious agents impacts offspring neurodevelopment. This latter aspect will be important to evaluate in future studies. This study provides an exciting hypothesis-generating set of preliminary data that will contribute to future studies addressing the interactions between autoimmunity and the impacts of infection during pregnancy.

## Data availability statement

The raw data supporting the conclusions of this article will be made available by the authors, without undue reservation.

## Ethics statement

The studies involving humans were approved by Institutional Review Board at Kaiser Permanente, the State Committee for the Protection of Human Subjects, and the Institutional Review Board at University of California, Davis. The studies were conducted in accordance with the local legislation and institutional requirements. Written informed consent for participation was not required from the participants or the participants’ legal guardians/next of kin because we used only banked samples that were exempt from informed consent.

## Author contributions

JM: Conceptualization, Formal analysis, Writing – original draft. LC: Conceptualization, Funding acquisition, Investigation, Resources, Writing – review & editing. AI: Formal analysis, Validation, Writing – review & editing. CY: Data curation, Methodology, Writing – review & editing. PA: Writing – review & editing. RY: Data curation, Methodology, Writing – review & editing, Resources. JV: Conceptualization, Data curation, Funding acquisition, Resources, Writing – review & editing.
