# Supplementary material for: Altered cytokine and chemokine profile linked to autoantibody and pathogen reactivity in mothers of autistic children
Source: Front Psychiatry. 2024 May 22;15:1348092. doi: 10.3389/fpsyt.2024.1348092 (PMC11150845; doi:10.3389/fpsyt.2024.1348092)
Supplement: Supplementary file 1 [file Table_1.docx]

Supplementary Table 1. Summary statistics including the mean (SD), lower (Q1), and upper (Q3) quartiles for cytokines and chemokines (μg/l) for MAR+ and MAR- mothers.

| Cytokine/Chemokine | MAR+  (n = 37) | | | MAR-  (n = 37) | | |
| --- | --- | --- | --- | --- | --- | --- |
|  | Mean (SD) | Q1 | Q3 | Mean (SD) | Q1 | Q3 |
| Eotaxin | 15.1 (25.1) | 2.6 | 17.2 | 161.1 (925.8) | 1.2 | 13.7 |
| GM-CSF | 25.5 (100.9) | 0.2 | 4.9 | 14.2 (59.8) | 0.1 | 5.5 |
| IFNγ | 36.6 (128.3) | 2.8 | 11.1 | 7.1 (16.8) | 1.7 | 7.6 |
| IL-2 | 4.0 (13.0) | 0.1 | 0.8 | 0.5 (0.6) | 0.1 | 0.6 |
| IL-4 | 500.4 (393.3) | 203.7 | 689.2 | 484.8 (300.1) | 289.0 | 628.3 |
| IL-6 | 187.5 (335.7) | 5.9 | 170.2 | 230.3 (640.8) | 1.8 | 97.0 |
| IL-7 | 9.5 (9.2) | 3.5 | 11.4 | 9.2 (10.3) | 3.1 | 9.9 |
| IL-8 | 2765 (2766) | 648.8 | 4801 | 2636 (2720) | 463.7 | 4313 |
| IL-10 | 32.8 (99.6) | 1.7 | 15.2 | 9.2 (17.8) | 1.2 | 8.4 |
| IL-13 | 9.7 (39.2) | 0.1 | 3.3 | 3.7 (5.3) | 0.1 | 6.1 |
| IL-17 | 4.7 (12.1) | 0.03 | 4.4 | 1.4 (2.6) | 0.1 | 1.3 |
| IL-12p40 | 35.8 (112.6) | 1.8 | 20.1 | 12.4 (23.9) | 1.1 | 7.9 |
| IL-12p70 | 7.5 (15.3) | 0.6 | 4.8 | 1.6 (1.7) | 0.3 | 2.2 |
| IL-1α | 16.1 (59.7) | 0.4 | 5.1 | 5.5 (11.4) | 0.2 | 7.4 |
| IL-1β | 207.6 (447.9) | 4.4 | 96.1 | 473.8 (2200) | 2.4 | 43.0 |
| IL-1Ra | 63.8 (123.6) | 11.1 | 55.2 | 37.1 (70.8) | 6.6 | 32.5 |
| IP-10 | 125.4 (184.7) | 41.0 | 106.4 | 98.0 (79.1) | 45.2 | 114.4 |
| MCP-1 | 691.0 (998.4) | 237.9 | 580.8 | 624.4 (854.2) | 255.4 | 533.7 |
| MIP-1α | 598.4 (1521.4) | 11.8 | 274.3 | 3726 (20246) | 10.9 | 351.6 |
| MIP-1β | 100.6 (160.7) | 15.6 | 95.8 | 248.1 (1070) | 11.1 | 53.7 |
| TNFα | 13.1(19.1) | 4.1 | 13.3 | 15.5 (36.0) | 2.2 | 10.9 |
| sIL-2Ra | 36.6 (28.0) | 17.5 | 51.5 | 33.1 (28.4) | 10.1 | 48.6 |

Abbreviations: MAR+: mothers with MAR-autism specific patterns of autoantibodies; MAR-: mothers without autoantibodies to any of our tested antigens; SD: standard deviation.

Supplementary Table 2. Summary statistics including the mean (SD), lower (Q1), and upper (Q3) quartiles for cytokines and chemokines (μg/l) for MAR+ and MAR- mothers who tested positive for CMV.

| Cytokine/Chemokine | MAR+  (n = 12) | | | MAR-  (n = 10) | | |
| --- | --- | --- | --- | --- | --- | --- |
|  | Mean (SD) | Q1 | Q3 | Mean (SD) | Q1 | Q3 |
| Eotaxin | 22.9 (38.2) | 6.5 | 22.6 | 4.9 (4.6) | 1.0 | 9.4 |
| GM-CSF | 70.2 (173.3) | 0.1 | 10.3 | 37.0 (115.1) | 0.1 | 1.4 |
| IFNγ | 8.4 (6.3) | 3.7 | 13.9 | 5.7 (4.2) | 2.0 | 9.6 |
| IL-2 | 6.2 (19.3) | 0.3 | 1.1 | 0.4 (0.5) | 0.1 | 0.5 |
| IL-4 | 594.8 (264.3) | 392.8 | 813.9 | 601.3 (350.7) | 368.7 | 908.8 |
| IL-6 | 236.6 (400.9) | 2.4 | 292.0 | 264.7 (692.2) | 8.9 | 102.1 |
| IL-7 | 9.8 (9.9) | 5.9 | 10.8 | 6.8 (6.1) | 3.1 | 6.9 |
| IL-8 | 3468 (3310) | 1352 | 5629 | 3310 (2448) | 1054 | 5026 |
| IL-10 | 72.1 (169.8) | 0.3 | 35.3 | 12.5 (30.7) | 0.2 | 8.1 |
| IL-13 | 3.5 (4.9) | 0.2 | 4.1 | 4.0 (6.7) | 0.1 | 5.3 |
| IL-17 | 1.5 (2.1) | 0.04 | 3.0 | 0.5 (0.6) | 0.02 | 1.0 |
| IL-12p40 | 74.8 (194.6) | 1.3 | 30.0 | 6.5 (11.6) | 1.4 | 6.0 |
| IL-12p70 | 5.9 (14.5) | 0.2 | 2.9 | 1.5 (1.4) | 0.6 | 2.1 |
| IL-1α | 9.1 (18.1) | 0.9 | 4.9 | 8.9 (20.4) | 0.4 | 4.0 |
| IL-1β | 263.8 (500.0) | 7.0 | 329.3 | 1342 (4187) | 3.2 | 35.3 |
| IL-1Ra | 111.2 (203.1) | 10.6 | 107.7 | 53.5 (123.5) | 7.8 | 23.4 |
| IP-10 | 115.3 (113.5) | 47.2 | 118.9 | 91.9 (58.9) | 44.3 | 126,2 |
| MCP-1 | 608.6 (945.2) | 236.5 | 514.3 | 645.3 (2051) | 249.3 | 556.8 |
| MIP-1α | 597.1 (1440) | 17.2 | 225.6 | 12428 (38984) | 30.0 | 249.0 |
| MIP-1β | 116.6 (153.2) | 16.3 | 211.6 | 675.9 (2051) | 14.5 | 45.3 |
| TNFα | 14.1 (23.7) | 2.6 | 13.3 | 24.4 (60.4) | 3.3 | 5.5 |
| sIL-2Ra | 33.8 (26.4) | 11.1 | 52.3 | 23.2 (22.4) | 4.3 | 33.9 |

Abbreviations: MAR+: mothers with MAR-autism specific patterns of autoantibodies; MAR-: mothers without autoantibodies to any of our tested antigens; CMV: cytomegalovirus; SD: standard deviation.

Supplementary Table 3. Summary statistics including the mean (SD), lower (Q1), and upper (Q3) quartiles for cytokines and chemokines (μg/l) for MAR+ and MAR- mothers who tested positive for EBV.

| Cytokine/Chemokine | MAR+  (n = 9) | | | MAR-  (n = 7) | | |
| --- | --- | --- | --- | --- | --- | --- |
|  | Mean (SD) | Q1 | Q3 | Mean (SD) | Q1 | Q3 |
| Eotaxin | 14.0 (8.8) | 9.9 | 22.0 | 10.7 (8.6) | 3.2 | 13.7 |
| GM-CSF | 28.2 (80.9) | 0.3 | 1.9 | 52.6 (137.6) | 0.1 | 2.9 |
| IFNγ | 9.4 (5.6) | 5.8 | 11.9 | 2.9 (1.4) | 2.0 | 4.1 |
| IL-2 | 0.5 (0.5) | 0.1 | 0.8 | 0.5 (0.4) | 0.1 | 0.8 |
| IL-4 | 430.0 (279.1) | 316.7 | 579.1 | 429.4 (278.9) | 188.9 | 486.4 |
| IL-6 | 189.4 (362.7) | 6.4 | 89.7 | 325.0 (840.5) | 1.1 | 23.6 |
| IL-7 | 13.7 (12.5) | 4.7 | 18.4 | 10.6 (15.0) | 3.6 | 6.6 |
| IL-8 | 3171 (2801) | 423.8 | 5662 | 2135 (2107) | 138.0 | 4313 |
| IL-10 | 38.2 (66.5) | 0.4 | 58.5 | 18.4 (36.7) | 0.3 | 23.6 |
| IL-13 | 6.1 (5.7) | 2.1 | 8.5 | 2.3 (5.5) | 0.1 | 1.0 |
| IL-17 | 1.5 (2.1) | 0.02 | 1.7 | 0.5 (0.7) | 0.02 | 0.8 |
| IL-12p40 | 33.1 (41.6) | 4.4 | 46.9 | 19.4 (40.3) | 1.7 | 11.1 |
| IL-12p70 | 7.1 (16.5) | 0.7 | 2.5 | 2.2 (1.9) | 0.7 | 3.5 |
| IL-1α | 11.8 (20.4) | 2.4 | 5.4 | 13.2 (24.0) | 0.2 | 15.3 |
| IL-1β | 209.9 (545.5) | 9.0 | 62.5 | 1899 (5008) | 1.6 | 21.5 |
| IL-1Ra | 76.1 (92.7) | 19.7 | 55.2 | 82.4 (149.3) | 4.0 | 133.8 |
| IP-10 | 70.3 (24.8) | 54.2 | 92.6 | 76.4 (34.3) | 54.4 | 114.2 |
| MCP-1 | 753.0 (1074) | 237.9 | 615.9 | 712.3 (1177) | 231.2 | 336.3 |
| MIP-1α | 791.6 (1639) | 11.8 | 274.3 | 17646 (46624) | 9.3 | 48.4 |
| MIP-1β | 95.5 (145.7) | 21.5 | 52.1 | 944.4 (2456) | 6.8 | 31.8 |
| TNFα | 15.6 (27.4) | 2.6 | 12.0 | 31.5 (72.5) | 2.8 | 10.9 |
| sIL-2Ra | 46.6 (34.6) | 17.6 | 80.9 | 19.3 (14.0) | 4.8 | 32.3 |

Abbreviations: MAR+: mothers with MAR-autism specific patterns of autoantibodies; MAR-: mothers without autoantibodies to any of our tested antigens. EBV: Epstein-Barr virus; SD: standard deviation.

Supplementary Table 4. Summary statistics including the mean (SD), lower (Q1), and upper (Q3) quartiles for cytokines and chemokines (μg/l) for MAR+ and MAR- mothers who tested positive for Flu-A.

| Cytokine/Chemokine | MAR+  (n = 14) | | | MAR-  (n = 7) | | |
| --- | --- | --- | --- | --- | --- | --- |
|  | Mean (SD) | Q1 | Q3 | Mean (SD) | Q1 | Q3 |
| Eotaxin | 23.9 (36.5) | 5.4 | 23.2 | 3.1 (4.9) | 0.5 | 2.4 |
| GM-CSF | 44.7 (152.5) | 0.1 | 11.2 | 1.1 (2.0) | 0.1 | 0.8 |
| IFNγ | 15.5 (23.0) | 3.5 | 16.0 | 2.6 (2.9) | 0.3 | 2.8 |
| IL-2 | 8.7 (20.2) | 0.3 | 2.1 | 0.2 (0.2) | 0.1 | 0.5 |
| IL-4 | 507.1 (244.0) | 316.7 | 692.0 | 432.8 (368.6) | 119.0 | 567.3 |
| IL-6 | 194.6 (357.9) | 2.5 | 121.6 | 41.7 (44.1) | 2.7 | 78.6 |
| IL-7 | 7.2 (3.7) | 4.7 | 9.9 | 11.3 (13.5) | 1.2 | 20.3 |
| IL-8 | 2685 (3368) | 262.9 | 2984 | 2536 (2991) | 197.3 | 6765 |
| IL-10 | 48.4 (152.9) | 1.6 | 11.9 | 8.8 (14.8) | 0.8 | 8.1 |
| IL-13 | 2.2 (2.7) | 0.1 | 3.0 | 3.4 (5.1) | 0.1 | 5.3 |
| IL-17 | 2.3 (3.3) | 0.1 | 4.4 | 0.3 (0.7) | 0.01 | 0.1 |
| IL-12p40 | 56.6 (180.2) | 1.5 | 13.0 | 18.1 (29.4) | 0.2 | 38.5 |
| IL-12p70 | 5.4 (10.3) | 0.2 | 3.4 | 0.9 (1.0) | 0.2 | 1.7 |
| IL-1α | 3.7 (3.6) | 0.3 | 5.4 | 3.5 (4.9) | 0.2 | 7.4 |
| IL-1β | 153.8 (274.5) | 2.6 | 83.2 | 25.4 (29.8) | 2.3 | 35.3 |
| IL-1Ra | 75.7 (181.3) | 11.1 | 47.2 | 24.8 (25.3) | 7.8 | 37.2 |
| IP-10 | 154.3 (224.7) | 38.6 | 151.6 | 142.2 (122.3) | 42.1 | 194.3 |
| MCP-1 | 483.5 (635.0) | 205.6 | 388.3 | 401.9 (207.6) | 255.4 | 574.7 |
| MIP-1α | 789.8 (2108) | 3.9 | 177.0 | 38.8 (47.8) | 4.4 | 49.1 |
| MIP-1β | 134.4 (229.2) | 14.2 | 187.8 | 15.9 (9.7) | 7.1 | 26.5 |
| TNFα | 8.9 (8.2) | 1.7 | 12.5 | 4.7 (3.2) | 1.3 | 5.5 |
| sIL-2Ra | 33.9 (32.7) | 17.6 | 42.9 | 35.5 (28.4) | 2.5 | 65.8 |

Abbreviations: MAR+: mothers with MAR-autism specific patterns of autoantibodies; MAR-: mothers without autoantibodies to any of our tested antigens. Flu-A: Influenza A virus, SD: standard deviation.

Supplementary Table 5. Summary statistics including the mean (SD), lower (Q1), and upper (Q3) quartiles for cytokines and chemokines (μg/l) for MAR+ and MAR- mothers who tested positive for VSV.

| Cytokine/Chemokine | MAR+  (n = 18) | | | MAR-  (n = 18) | | |
| --- | --- | --- | --- | --- | --- | --- |
|  | Mean (SD) | Q1 | Q3 | Mean (SD) | Q1 | Q3 |
| Eotaxin | 20.8 (33.0) | 2.2 | 23.2 | 12.6 (10.7) | 3.6 | 17.8 |
| GM-CSF | 35.6 (134.5) | 0.2 | 11.2 | 5.1 (10.1) | 0.1 | 5.5 |
| IFNγ | 14.5 (21.4) | 2.5 | 16.0 | 11.3 (23.5) | 2.0 | 9.6 |
| IL-2 | 6.9 (18.1) | 0.05 | 1.5 | 0.6 (0.5) | 0.1 | 0.8 |
| IL-4 | 521.0 (460.1) | 226.4 | 811.3 | 473.0 (252.8) | 304.6 | 628.3 |
| IL-6 | 163.3 (319.9) | 1.7 | 89.7 | 71.7 (131.6) | 2.5 | 97.0 |
| IL-7 | 9.8 (10.6) | 3.1 | 11.5 | 9.2 (8.5) | 3.3 | 15.3 |
| IL-8 | 2578 (2355) | 151.4 | 3031 | 2693 (2624) | 606.5 | 3732 |
| IL-10 | 11.9 (19.4) | 1.3 | 6.9 | 5.3 (7.4) | 0.8 | 5.3 |
| IL-13 | 3.0 (4.7) | 0.1 | 3.0 | 3.9 (5.8) | 0.1 | 6.2 |
| IL-17 | 2.1 (3.2) | 0.03 | 4.4 | 1.4 (2.1) | 0.03 | 2.0 |
| IL-12p40 | 51.3 (160.4) | 0.9 | 13.0 | 9.2 (16.1) | 1.1 | 6.0 |
| IL-12p70 | 7.4 (13.2) | 0.3 | 7.9 | 1.5 (1.4) | 0.3 | 2.1 |
| IL-1α | 3.7 (5.7) | 0.3 | 3.8 | 3.5 (5.3) | 0.2 | 4.0 |
| IL-1β | 130.0 (245.2) | 2.6 | 83.9 | 38.8 (73.7) | 3.2 | 26.8 |
| IL-1Ra | 81.4 (162.6) | 6.7 | 59.9 | 18.4 (19.4) | 6.6 | 22.1 |
| IP-10 | 185.4 (251.8) | 48.6 | 151.6 | 114.1 (101.0) | 54.6 | 114.4 |
| MCP-1 | 720.1 (1133) | 239.7 | 523.6 | 671.3 (975.1) | 248.8 | 533.7 |
| MIP-1α | 741.5 (1858) | 5.2 | 352.4 | 185.9 (251.9) | 11.7 | 351.6 |
| MIP-1β | 133.3 (202.9) | 17.0 | 187.8 | 40.3 (40.7) | 15.1 | 53.7 |
| TNFα | 8.8 (7.8) | 1.7 | 12.5 | 7.6 (8.3) | 2.2 | 10.9 |
| sIL-2Ra | 36.4 (34.8) | 11.6 | 53.1 | 28.9 (23.4) | 6.3 | 37.7 |

Abbreviations: MAR+: mothers with MAR-autism specific patterns of autoantibodies; MAR-: mothers without autoantibodies to any of our tested antigens. VSV: Varicella-Zoster Virus.

Supplementary Table 6. Summary statistics including the mean (SD), lower (Q1), and upper (Q3) quartiles for cytokines and chemokines (μg/l) for MAR+ and MAR- mothers who tested positive for HSV.

| Cytokine/Chemokine | MAR+  (n = 26) | | | MAR-  (n = 21) | | |
| --- | --- | --- | --- | --- | --- | --- |
|  | Mean (SD) | Q1 | Q3 | Mean (SD) | Q1 | Q3 |
| Eotaxin | 15.6 (28.3) | 2.2 | 17.7 | 277.1 (1228) | 1.6 | 10.3 |
| GM-CSF | 35.4 (119.7) | 0.2 | 11.2 | 23.4 (78.9) | 0.1 | 9.1 |
| IFNγ | 19.1 (49.0) | 2.5 | 11.9 | 3.3 (3.2) | 0.8 | 4.8 |
| IL-2 | 4.7 (15.2) | 0.1 | 0.8 | 0.4 (0.7) | 0.1 | 0.6 |
| IL-4 | 481.1 (406.2) | 203.7 | 676.1 | 541.3 (339.9) | 289.0 | 711.1 |
| IL-6 | 246.8 (382.6) | 6.4 | 202.4 | 384.4 (824.8) | 1.0 | 214 |
| IL-7 | 8.4 (9.6) | 2.3 | 10.2 | 6.8 (7.9) | 2.1 | 7.1 |
| IL-8 | 3266 (2915) | 948.4 | 5596 | 3359 (3230) | 245.1 | 6764 |
| IL-10 | 17.4 (41.2) | 1.7 | 11.9 | 9.7 (21.4) | 0.4 | 8.3 |
| IL-13 | 2.8 (4.5) | 0.04 | 3.2 | 2.3 (3.0) | 0.1 | 4.7 |
| IL-17 | 3.3 (7.2) | 0.03 | 4.4 | 1.3 (2.9) | 0.1 | 0.9 |
| IL-12p40 | 41.8 (133.7) | 1.5 | 13.0 | 8.1 (11.7) | 0.6 | 7.9 |
| IL-12p70 | 4.6 (10.8) | 0.3 | 2.8 | 1.2 (1.4) | 0.4 | 1.6 |
| IL-1α | 7.4 (15.7) | 0.9 | 4.4 | 6.8 (14.3) | 0.4 | 7.4 |
| IL-1β | 283.8 (517.7) | 4.4 | 259.0 | 821.6 (2901) | 2.1 | 86.5 |
| IL-1Ra | 75.1 (143.4) | 11.1 | 59.9 | 46.2 (88.7) | 6.6 | 37.2 |
| IP-10 | 119.3 (165.6) | 41.0 | 115.7 | 91.8 (82.1) | 38.5 | 114.2 |
| MCP-1 | 819.4 (1167) | 239.7 | 580.8 | 826.6 (1096) | 255.4 | 608.1 |
| MIP-1α | 783.7 (1777) | 12.8 | 387.8 | 6479 (26822) | 4.4 | 609.2 |
| MIP-1β | 125.9 (183.7) | 20.9 | 171.7 | 409.9 (1413) | 7.1 | 75.7 |
| TNFα | 14.8 (22.2) | 3.1 | 15.4 | 23.6 (46.5) | 1.5 | 17.7 |
| sIL-2Ra | 30.5 (23.1) | 12.7 | 45.5 | 24.1 (20.6) | 6.3 | 35.0 |

Abbreviations: MAR+: mothers with MAR-autism specific patterns of autoantibodies; MAR-: mothers without autoantibodies to any of our tested antigens; HSV: Herpes Simplex Virus; SD: standard deviation.

Supplementary Table 7. Summary statistics including the mean (SD), lower (Q1), and upper (Q3) quartiles for cytokines and chemokines (μg/l) for MAR+ and MAR- mothers who tested positive for TOXO.

| Cytokine/Chemokine | MAR+  (n = 3) | | | MAR-  (n = 3) | | |
| --- | --- | --- | --- | --- | --- | --- |
|  | Mean (SD) | Q1 | Q3 | Mean (SD) | Q1 | Q3 |
| Eotaxin | 7.8 (6.1) | 0.8 | 11.5 | 25.8 (15.7) | 9.4 | 40.7 |
| GM-CSF | 5.8 (5.4) | 1.0 | 11.6 | 4.6 (3.4) | 2.4 | 8.5 |
| IFNγ | 85.4 (140.2) | 1.3 | 247.2 | 6.3 (2.9) | 4.1 | 9.6 |
| IL-2 | 0.2 (0.2) | 0.04 | 0.43 | 0.5 (0.3) | 0.4 | 0.8 |
| IL-4 | 467.0 (282.4) | 145.8 | 676.1 | 409.7 (338.6) | 188.9 | 799.5 |
| IL-6 | 106.5 (56.1) | 64.5 | 170.2 | 106.7 (97.3) | 23.6 | 213.8 |
| IL-7 | 3.6 (4.8) | 0.03 | 9.1 | 14.0 (16.6) | 3.1 | 33.1 |
| IL-8 | 5836 (614.9) | 5327 | 6519 | 5890 (3574) | 3552 | 10004 |
| IL-10 | 7.1 (4.2) | 4.1 | 11.9 | 5.5 (6.9) | 0.1 | 13.2 |
| IL-13 | 2.9 (4.9) | 0.03 | 8.5 | 2.2 (3.5) | 0.1 | 6.2 |
| IL-17 | 0.4 (0.7) | 0.01 | 1.2 | 2.1 (1.2) | 1.0 | 3.4 |
| IL-12p40 | 36.9 (29.1) | 4.1 | 59.8 | 11.2 (10.7) | 0.6 | 21.9 |
| IL-12p70 | 5.1 (7.8) | 0.2 | 14.1 | 2.4 (1.0) | 1.5 | 3.5 |
| IL-1α | 3.9 (1.3) | 2.9 | 5.4 | 9.3 (5.7) | 4.0 | 15.3 |
| IL-1β | 89.9 (32.7) | 61.1 | 125.4 | 55.4 (32.3) | 21.5 | 85.8 |
| IL-1Ra | 79.4 (88.9) | 11.1 | 179.9 | 32.3 (33.3) | 10.6 | 70.6 |
| IP-10 | 59.4 (48.8) | 28.6 | 115.7 | 97.9 (17.4) | 78.1 | 110.8 |
| MCP-1 | 1727 (2558) | 231.0 | 4681 | 1446 (2125) | 205.9 | 3901 |
| MIP-1α | 263.1 (9.8) | 256.3 | 274.3 | 282.8 (225.6) | 23.1 | 430.4 |
| MIP-1β | 57.3 (36.6) | 22.9 | 95.8 | 36.2 (22.4) | 11.1 | 54.1 |
| TNFα | 8.2 (4.6) | 4.6 | 13.3 | 20.0 (10.4) | 10.9 | 31.4 |
| sIL-2Ra | 30.8 (28.5) | 11.3 | 63.5 | 13.9 (10.0) | 6.3 | 25.3 |

Abbreviations: MAR+: mothers with MAR-autism specific patterns of autoantibodies; MAR-: mothers without autoantibodies to any of our tested antigens; TOXO: Toxoplasmosis; SD: standard deviation.

Supplementary Table 8. Summary statistics including the mean (SD), lower (Q1), and upper (Q3) quartiles for cytokines and chemokines (μg/l) for MAR+ and MAR- mothers who tested positive for CRP.

| Cytokine/Chemokine | MAR+  (n =10) | | | MAR-  (n =12) | | |
| --- | --- | --- | --- | --- | --- | --- |
|  | Mean (SD) | Q1 | Q3 | Mean (SD) | Q1 | Q3 |
| Eotaxin | 29.2 (43.2) | 6.3 | 28.1 | 7.4 (11.8) | 0.6 | 10.6 |
| GM-CSF | 59.8 (180.7) | 0.02 | 9.2 | 4.5 (12.0) | 0.1 | 1.0 |
| IFNγ | 79.0 (237.2) | 1.5 | 7.6 | 5.4 (4.0) | 2.4 | 9.2 |
| IL-2 | 9.0 (21.2) | 0.4 | 1.5 | 0.4 (0.6) | 0.1 | 0.5 |
| IL-4 | 478.1 (253.3) | 203.7 | 692.0 | 510.6 (334.8) | 211.8 | 713.9 |
| IL-6 | 91.2 (157.2) | 2.3 | 89.7 | 91.3 (158.1) | 2.2 | 110.6 |
| IL-7 | 13.6 (13.2) | 4.2 | 22.1 | 12.5 (12.5) | 3.1 | 19.8 |
| IL-8 | 2626 (2424) | 731.3 | 4800 | 2373 (3183) | 330.5 | 2854 |
| IL-10 | 21.1 (33.5) | 1.7 | 23.3 | 10.5 (12.9) | 1.2 | 12.7 |
| IL-13 | 28.7 (74.4) | 1.3 | 11.0 | 5.2 (6.7) | 0.1 | 7.6 |
| IL-17 | 7.0 (20.7) | 0.03 | 1.1 | 0.9 (1.1) | 0.02 | 1.5 |
| IL-12p40 | 98.2 (208.9) | 4.0 | 60.9 | 17.7 (26.9) | 0.2 | 30.2 |
| IL-12p70 | 14.6 (25.3) | 0.3 | 10.3 | 1.7 (1.8) | 0.3 | 2.4 |
| IL-1α | 39.8 (112.8) | 0.3 | 5.4 | 2.7 (4.0) | 0.2 | 2.4 |
| IL-1β | 120.9 (236.4) | 2.0 | 96.1 | 55.4 (88.3) | 2.7 | 86.1 |
| IL-1Ra | 123.4 (210.9) | 12.7 | 160.1 | 29.8 (25.9) | 8.5 | 48.7 |
| IP-10 | 182.4 (253.5) | 50.6 | 116.8 | 91.8 (85.7) | 43.7 | 112.6 |
| MCP-1 | 393.4 (235.1) | 237.9 | 523.6 | 877.3 (1151) | 301.7 | 582.5 |
| MIP-1α | 333.6 (794.1) | 12.8 | 203.7 | 148.7 (245.4) | 11.3 | 194.4 |
| MIP-1β | 89.0 (124.8) | 21.5 | 67.2 | 28.6 (22.4) | 12.5 | 44.4 |
| TNFα | 6.5 (5.7) | 2.2 | 8.9 | 8.0 (9.0) | 3.5 | 7.8 |
| sIL-2Ra | 42.0 (27.3) | 12.8 | 63.5 | 38.2 (35.4) | 8.2 | 61.5 |

Abbreviations: MAR+: mothers with MAR-autism specific patterns of autoantibodies; MAR-: mothers without autoantibodies to any of our tested antigens; CRP: C-reactive protein; SD: standard deviation.
